# ProbeDealer is a convenient tool for designing probes for highly multiplexed fluorescence in situ hybridization

**DOI:** 10.1038/s41598-020-76439-x

**Published:** 2020-12-16

**Authors:** Mengwei Hu, Bing Yang, Yubao Cheng, Jonathan S. D. Radda, Yanbo Chen, Miao Liu, Siyuan Wang

**Affiliations:** 1 Department of Genetics, Yale School of Medicine, Yale University, New Haven, CT 06510 USA; 2 Department of Cell Biology, Yale School of Medicine, Yale University, New Haven, CT 06510 USA; 3 Yale Combined Program in the Biological and Biomedical Sciences, Yale University School of Medicine, New Haven, CT USA; 4 Molecular Cell Biology, Genetics and Development Program, Yale University School of Medicine, New Haven, CT USA; 5 Biochemistry, Quantitative Biology, Biophysics and Structural Biology Program, Yale University School of Medicine, New Haven, CT USA; 6 M.D.-Ph.D. Program, Yale University School of Medicine, New Haven, CT USA; 7 Yale Center for RNA Science and Medicine, Yale University School of Medicine, New Haven, CT USA; 8 Yale Liver Center, Yale University School of Medicine, New Haven, CT USA

**Keywords:** Imaging, Software, Oligonucleotide probes

## Abstract

Fluorescence in situ hybridization (FISH) is a powerful method to visualize the spatial positions of specific genomic loci and RNA species. Recent technological advances have leveraged FISH to visualize these features in a highly multiplexed manner. Notable examples include chromatin tracing, RNA multiplexed error-robust FISH (MERFISH), multiplexed imaging of nucleome architectures (MINA), and sequential single-molecule RNA FISH. However, one obstacle to the broad adoption of these methods is the complexity of the multiplexed FISH probe design. In this paper, we introduce an easy-to-use, versatile, and all-in-one application called ProbeDealer to design probes for a variety of multiplexed FISH techniques and their combinations. ProbeDealer offers a one-stop shop for multiplexed FISH design needs of the research community.

## Introduction

Fluorescence in situ hybridization (FISH) can be used to visualize spatial locations of DNA regions and RNA molecules in a sequence-specific manner. Recently, FISH has been extended with multiplicity to profile chromatin folding pattern and the abundance of numerous transcripts in several methods. These include multiplexed sequential DNA FISH^1–6^ (termed chromatin tracing), multiplexed error-robust FISH (MERFISH)^7–10^ and similar methods^11,12^, and sequential single-molecule RNA FISH (sequentially imaging individual RNA species without combinatorial barcoding)^4,9^. Chromatin tracing has been combined with single-molecule RNA FISH to study the association between gene expression regulation and chromatin folding^4,5^. Recently, our group reported multiplexed imaging of nucleome architectures (MINA)^13^, a method that combines chromatin tracing, RNA MERFISH and protein labeling. We used MERFISH and cell boundary labeling to distinguish different cell types in a highly heterogeneous mammalian tissue, and used chromatin tracing and co-immunofluorescence to profile three-dimensional genomic architectures in single nuclei across different length scales and in relation to other nuclear components^13^. These FISH-based methods greatly advanced the characterization of spatial-omics and their physiological relevance. However, one technical obstacle to the broad adaption of these highly multiplexed techniques is their complex probe design procedure. Here, we introduce ProbeDealer, an easy-to-use application that facilitates probe design for chromatin tracing, RNA MERFISH and sequential single-molecule RNA FISH of individual transcript species.

Both chromatin tracing and RNA MERFISH use a two-stage hybridization procedure. In stage one, a library of oligonucleotide probes termed primary probes targeting all genomic loci or RNA species of interest are simultaneously hybridized to the targets. Each primary probe contains a targeting region that hybridizes to the target, and one or more overhanging readout regions. In stage two, dye-labeled secondary probes with different sequences complementing the readout regions on the primary probes are sequentially hybridized to the sample, imaged, and then bleached or removed. To design the primary probes with high hybridization efficiency and specificity, several requirements need to be met, including proper melting temperature (Tm), GC content, minimal secondary structure, minimal cross-hybridization between probes, and lack of long consecutive repeats of the same nucleotide^14^. These requirements were often fulfilled using OligoArray 2.1^15^ in previous works^1,7,13^, which requires familiarity with UNIX. Unfortunately, OligoArray 2.1 is no longer available for download. Thus, users without a previously installed copy of the software will not be able to use our published workflow. Even with OligoArray 2.1, probe design using our previous workflow can be challenging: Output probes must be filtered through additional rounds of BLAST^16^ to select specific probes, and must be extended with combinations of readout regions and priming sequences, which requires additional programming. The probe filtering and sequence extension algorithms depend on the probe type. Users need to make multiple changes to our previous MATLAB codes to adapt them for their needs. ProbeDealer simplifies this process by integrating probe design, BLAST, and other modifications into one program, and allows versatile probe design for several multiplexed FISH methods. Its graphical user interface improves user experience and eliminates the need for coding expertise.

## Results and discussion

### Generate primary probe sequences with customizable physical properties

**Figure 1:**
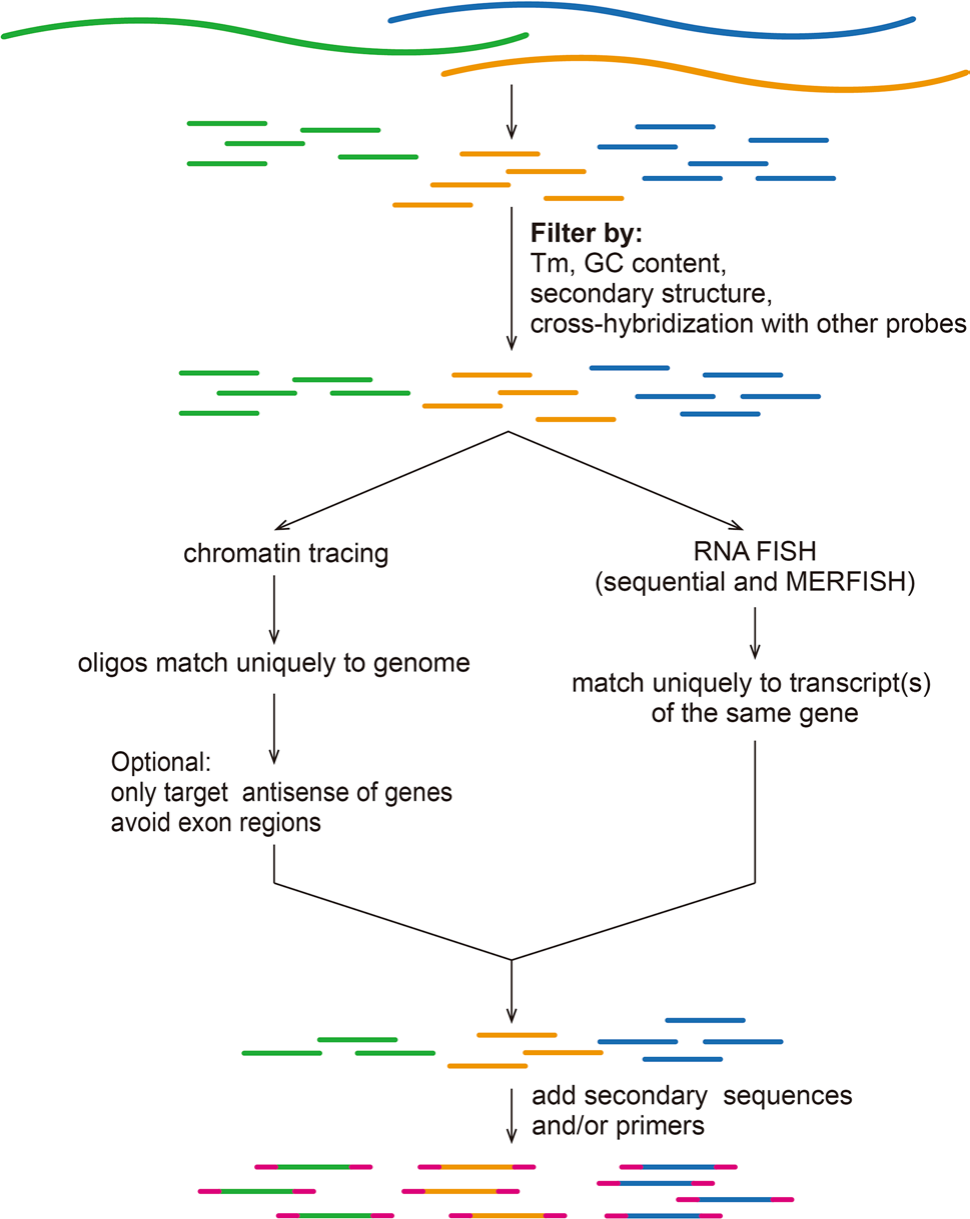
Probe design workflow. ProbeDealer scans input sequences to generate oligos of defined length, and filters the oligos according to their physical properties. ProbeDealer then examines the specificity of these probes, and outputs probes with appended priming regions for probe synthesis and secondary sequences for visualization.

The entire workflow of ProbeDealer is illustrated in Fig. 1, which includes three main steps: generate primary probes and filter them by their physical properties, filter primary probes by specificity, and generate outputs. To generate primary probes from target sequences (targeted genomic region or RNA sequences), ProbeDealer utilizes the following algorithm implemented in MATLAB: Each target sequence is scanned with a sliding window with customizable length (default is 30 nucleotides). For simplicity, the scanned sequences within the window, henceforth referred to as “oligos”, are on the same strand as the input sequences, and thus need to be reverse-complemented in a later step to generate probes that hybridize to the input sequences. Oligos are first filtered by GC content and repetitive nucleotides. Tm is calculated using the *oligoprop* function in the MATLAB Bioinformatics Toolbox. To avoid probes containing stable secondary structures, we apply the *rnafold* function to identify stem-loop structure on oligos. To reject probes that cross-hybridize with other probes, we perform local sequence alignment between each new candidate oligo and the currently accepted oligos using the *swalign* function. Of note, to facilitate the probe design process, we provide a set of default probe design parameters in an input Excel spreadsheet (oligoparameter.xlsx) which has been tested in our previous experiments^13^. Users may provide their customized probe design parameters by editing the Excel spreadsheet.

### Ensure probe specificity for multiple probe design scenarios

To ensure hybridization specificity, ProbeDealer then BLASTs accepted oligos against specific genomes and transcriptomes, depending on their intended application. For chromatin tracing, oligos are BLASTed against the whole genome in both directions to find oligos that only have one alignment in the genome. Because chromatin tracing is routinely performed with RNase treatment^1^, RNA will be eliminated and will not bind probes targeting the same sequences in the genome. To accommodate users who may need to retain RNAs, such as for MINA^13^, ProbeDealer offers an additional feature to design chromatin tracing probes targeting only the antisense strands of gene regions, and therefore even when transcripts are present, the probes will not hybridize to them. Towards this end, users may enable an “only target antisense strand” option in ProbeDealer to BLAST the oligos that passed the whole-genome BLAST further against the unspliced transcriptome in the plus/plus orientation. If an oligo (or its reverse complement) has no hits in the unspliced transcriptome, the oligo (or its reverse complement) is retained. If it is aligned to one or more entries of the unspliced transcriptome in the plus/plus direction, the probe (reverse complement of the oligo) will bind to the unspliced transcript(s), and thus this oligo is rejected.

In addition, to avoid chromatin tracing probes cross-hybridizing with RNA FISH probes applied to the same sample, ProbeDealer can optionally avoid exon regions of genes. This feature is important especially for MINA, when chromatin tracing and RNA FISH are combined and may target the DNA and RNA from the same genomic region. To achieve this, all chromatin tracing oligos are further BLASTed against spliced transcriptome. Oligos with alignment(s) to the spliced transcriptomes are rejected.

To design probes for multiplexed RNA FISH methods, ProbeDealer BLASTs oligos against the spliced transcriptome, and retain probes that only bind to transcript isoforms of the same gene. For both chromatin tracing and RNA FISH probes, users may choose to output all probe sequences, or define how many probes they want for each input target sequence.

### Export probe design outputs

After filtering oligos according to their physical properties and genomic and/or transcriptomic specificity, ProbeDealer appends sequences of the secondary probes and additional priming regions to the oligos to generate the final template oligos for probe synthesis. ProbeDealer provides 50 published default secondary probe sequences^13^ for chromatin tracing probes, and 16 published default secondary probe sequences^8^ for MERFISH and sequential single-molecule RNA FISH. Users may modify or add secondary probe sequences in the provided Excel spreadsheet of the ProbeDealer package.

We offer two choices of output format: (1) a template oligo library, which may be ordered as oligo pools for probe synthesis and amplification according to a previously established protocol (note the final primary probes are reverse-complements of template oligo sequences)^1,13^; (2) primary probe sequences, which can be ordered individually and used directly in primary hybridization. In the first option, three pairs of primer sequences are provided according to previous publications^1,13^, with one pair per probe type (chromatin tracing, RNA MERFISH, or sequential single-molecule RNA FISH), so users may combine multiple template oligo libraries from separate ProbeDealer outputs as sub-libraries in one oligo pool order, and selectively synthesize and amplify each sub-library. In the second option, priming regions are not added, and the primary probe sequences are computationally reverse-complemented from the template oligo sequences. This second option is particularly useful for designing small libraries of sequential single-molecule RNA FISH probes for individual targets.

### User interface

**Figure 2:**
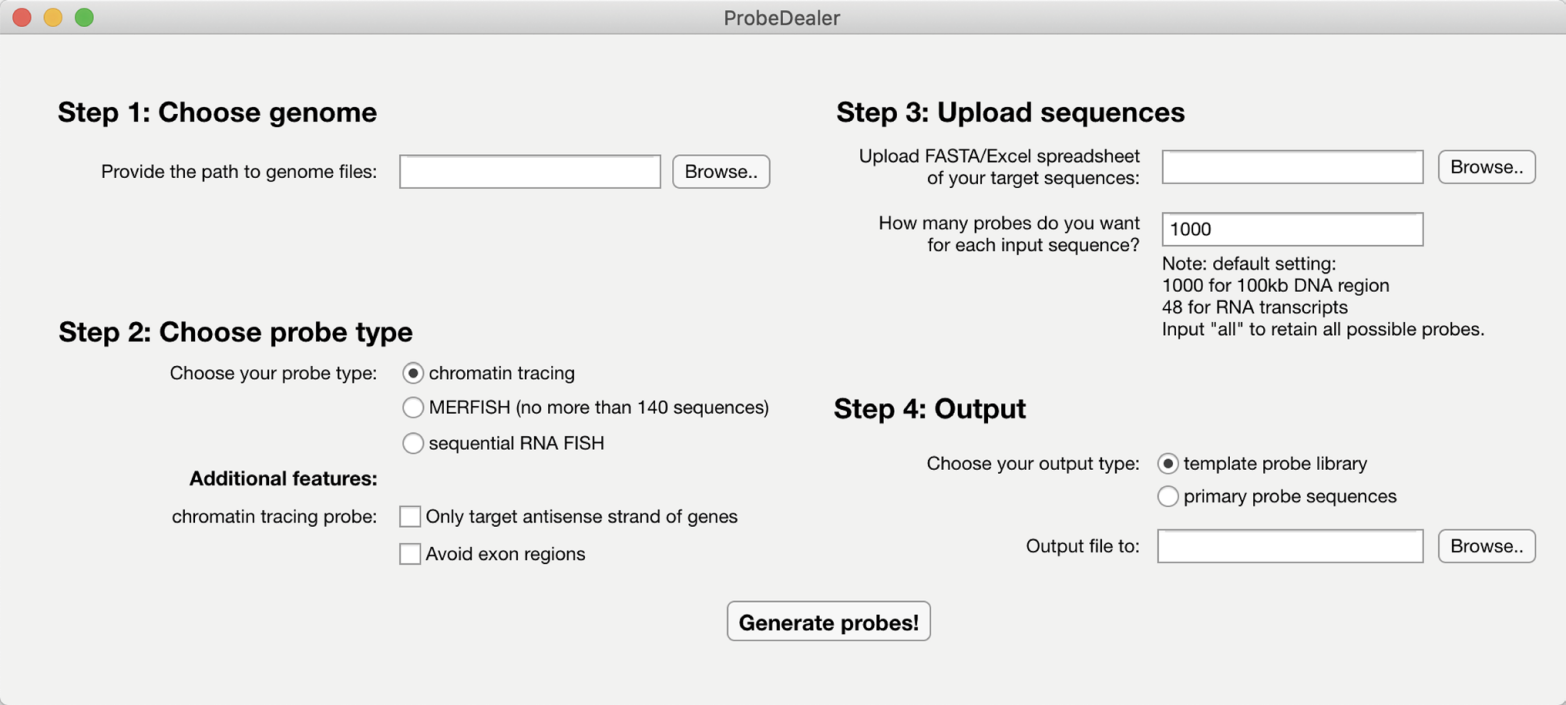
ProbeDealer user panel. ProbeDealer allows users to provide genome files and input target sequences. Users can specify their desired probe type, probe features, output type and path, and numbers of probes for each input sequence.

We have packaged ProbeDealer as a MATLAB application for Windows and Mac users, and a standalone application for Windows users. The user panel of ProbeDealer is shown in Fig. 2. We have provided complete human and mouse ProbeDealer BLAST databases using hg38 and mm10, respectively. Users may provide additional genome files for other species. To run ProbeDealer, users need to provide a fasta file of input target sequences, or a spreadsheet that (1) for chromatin tracing: contains the coordinates of the target genomic loci; (2) for RNA FISH: contains Ensembl transcript IDs of the target transcripts. For RNA MERFISH, only spreadsheet input files are accepted, and users should also provide bulk gene expression values (such as FPKM values from bulk RNA sequencing) in the spreadsheet. Using the gene expression values, ProbeDealer automatically rearranges the Modified-Hamming-Distance-4 (MHD4) codes^7^ used in MERFISH encoding to reduce bit sharing among highly expressed genes. The ProbeDealer output includes a fasta file and an Excel spreadsheet that contain the full list of template oligo or primary probe sequences. For MERFISH users, ProbeDealer also generates a code book matching MERFISH targets and MHD4 codes in an Excel spreadsheet. A detailed description of ProbeDealer’s internal probe design parameters and procedures is included in the Methods. A user manual including download links of ProbeDealer and examples is included in Supplementary Note S1. Instructions regarding dye-labeled secondary probes and primers for probe library amplification that are compatible with the ProbeDealer outputs are included in Supplementary Note S2.

### Time cost and computational resource usage of ProbeDealer

To estimate the time and memory usage for ProbeDealer to finish different probe design tasks, we recapitulated several probe design schemes in our previous publication^13^. Specifically, we performed three test runs using ProbeDealer on a workstation with 32 GB RAM using a single core: (1) designing topologically-associating-domain (TAD)-to-chromosome-scale chromatin tracing probes targeting the central 100-kb regions of 50 TADs on mouse chr19, (2) designing promoter-enhancer-scale chromatin tracing probes targeting 19 consecutive 5-kb regions upstream of gene *Scd2* on mouse chr19, and (3) designing RNA MERFISH probes targeting 136 mouse transcripts^13^. The TAD-to-chromosome-scale chromatin tracing design finished in 23.8 h and consumed 5.97 GB RAM. The fine-scale chromatin tracing and RNA MERFISH design took 6.3 min and 18.5 min, respectively, and consumed RAM of 5.47 GB and 1.74 GB, respectively.

We also tested TAD-to-chromosome-scale chromatin tracing design on human genome. Specifically, we recapitulated three chromatin tracing probe designs reported in a previous publication^1^: (1) designing a probe library targeting 30 TADs on human chr20, which takes 19.5 h; (2) designing a probe library targeting 34 TADs on human chr21, which takes 23.1 h; and (3) designing a probe library targeting 40 TADs on human chrX, which takes 21.3 h. The test runs above can also be performed on a personal laptop with 16 GB RAM.

### Comparison with other tools

Given the stringent probe specificity consideration in ProbeDealer, we set out to test whether ProbeDealer can yield sufficient number of probes, which is essential for good signal-to-background ratio especially in RNA FISH. We performed test runs of designing RNA MERFISH probes targeting 136 mouse transcripts probed in our previous work^13^ using three packages: ProbeDealer, OligoArray 2.1^15^ and OligoMiner^17^, and compared the number of probes generated by the packages. The probe count per kb of transcript is 29.3 for ProbeDealer, 18.7 for OligoArray 2.1 and 29.1 for OligoMiner. Therefore, ProbeDealer generates comparable number of probes as OligoMiner, which is higher than OligoArray 2.1. Thus, ProbeDealer and OligoMiner are more suitable for designing probes for shorter transcripts, which may not offer enough probes when OligoArray 2.1 is used. A detailed comparison of probe counts among ProbeDealer, OligoArray 2.1 and OligoMiner can be found in Supplementary Table S1.

We further compared these probe design tools in terms of their software purposes, computational resource requirements and time costs, probe design criteria, and user-friendliness in Supplementary Note S3. In summary, ProbeDealer is specialized in easy adaption and designing probes for multiple versions of multiplexed sequential FISH experiments, and generates comparable numbers of probes as other tools in relatively short time (Supplementary Note S3; Supplementary Table S1). With a graphical-user interface on a local computer, ProbeDealer is also more user-friendly and reliable than command-line-based probe design packages or online applications.

We hope ProbeDealer will simplify probe design for chromatin tracing, RNA MERFISH, sequential single-molecule RNA FISH and MINA, so these methods may be more easily utilized across the scientific community.

## Methods

### Prepare BLAST database

Human and mouse genomes are provided with ProbeDealer. All BLAST databases were generated by makeblastdb command in NCBI BLAST+^16^. The human genome fasta file (hg38) was downloaded from UCSC genome browser (https://hgdownload.soe.ucsc.edu/goldenPath/hg38/bigZips/hg38.fa.gz). The human transcriptome fasta file (Gencode v34) was downloaded from Gencode (ftp://ftp.ebi.ac.uk/pub/databases/gencode/Gencode_human/release_34/gencode.v34.transcripts.fa.gz), and the headers of transcript sequences were trimmed in MATLAB so they only contain Ensembl transcript ID without version variant information (e.g. ENST00000645576, ENSMUST00000106216). To generate the human unspliced transcriptome, the GTF file of hg38 was downloaded from the UCSC genome browser (https://hgdownload.soe.ucsc.edu/goldenPath/hg38/bigZips/genes/hg38.ensGene.gtf.gz), and coordinates of transcript start and end were extracted in MATLAB into a BED file. The human unspliced transcriptome fasta file was generated from the BED file and hg38 genome fasta file using command getfasta in BEDTools^18^.

The mouse genome fasta file (mm10) was downloaded from UCSC genome browser (https://hgdownload.soe.ucsc.edu/goldenPath/mm10/bigZips/mm10.fa.gz). The mouse transcriptome fasta file (Gencode vM25) was downloaded from Gencode (ftp://ftp.ebi.ac.uk/pub/databases/gencode/Gencode_mouse/release_M25/gencode.vM25.transcripts.fa.gz). The GTF file was downloaded from UCSC genome browser (https://hgdownload.soe.ucsc.edu/goldenPath/mm10/bigZips/genes/mm10.ensGene.gtf.gz). Mouse spliced and unspliced transcriptomes were prepared as described for the human transcriptome.

### Generate oligos from input sequences

Default probe design uses parameters reported in Ref.^13^, and requires the primary targeting region of probes to be 30-nucleotide (nt) in length, with melting temperature (Tm) not lower than 66 °C, and GC content between 30 and 90%. More than five repetitive nucleotides are excluded (e.g. GGGGGG). If probes form secondary structures, the Tm of concatenated stem sequence should not exceed 76 °C. If they cross-hybridize with other probes, the Tm of concatenated hybridized region should not exceed 72 °C. As recommended in OligoArray 2.0^15^, Tm is calculated via the nearest neighbor method^19^ under the assumption of 1 mol/liter salt concentration and 1e-6 mol/liter probe concentration. Input sequences are scanned with a 30-nt window from the 5′ end. If the current test oligo is accepted, the window moves 30 nt towards 3′ end to evaluate the next adjacent oligo; otherwise, the window moves 1 nt towards 3′ end for a new test oligo. These parameters can be customized by editing oligoparameter.xlsx in the ProbeDealer package.

### BLAST oligos

For chromatin tracing probes, oligos are first BLASTed against the genome, and only those with unique alignment in the genome are retained. If the “only target antisense of gene” feature is selected, retained oligos are additionally BLASTed against the unspliced transcriptome. Oligos without alignment to the unspliced transcriptome in plus/plus direction are retained. If an oligos is aligned to the unspliced transcriptome in the plus/plus direction but not in the plus/minus direction, its reverse-complement is retained as a qualified oligo. If the “avoid exon regions” feature is selected, qualified oligos are BLASTed once more against the spliced transcriptome. Probes with alignment to the spliced transcriptome are rejected.

For RNA FISH probes, including those for RNA MERFISH and sequential single-molecule RNA FISH, oligos are BLASTed against the transcriptome, and oligos matching the transcripts of multiple genes are rejected.

### Append secondary sequences and/or priming regions

For chromatin tracing and sequential single-molecule RNA FISH probes, 50 and 16 default secondary sequences that were previously validated^8,13^ are provided, respectively. These sequences are appended to the up- and down-stream of the primary targeting region of oligos so that oligos targeting the same input sequence share the same secondary sequence. If the number of input sequences for chromatin tracing or sequential single-molecule RNA FISH exceeds the number of provided secondary sequences, users should provide additional secondary sequences in the secondary sequence Excel spreadsheets (DNA secondaries.xlsx for chromatin tracing probes, RNA secondaries.xlsx for sequential single-molecule RNA FISH probes) in the ProbeDealer package.

For MERFISH probes, ProbeDealer provides the Modified-Hamming-Distance-4 (MHD4)^7^ coding scheme, which accepts up to 140 input sequences. The MHD4 codes are rearranged according to bulk gene expression values to avoid bit sharing among highly expressed genes. Each of the probes targeting one transcript carries three out of four secondary sequences that are assigned to that transcript, with one secondary sequence upstream of the primary targeting region of the oligo and two secondary sequences downstream.

If users want to order template oligos as a pooled library for primary probe synthesis and amplification^1,13^, priming regions are appended to both ends of the oligo sequences. By default, chromatin tracing probes, sequential single-molecule RNA FISH probes and MERFISH probes each have one distinct pair of priming sequences. The default priming sequences are stored in Primers.xlsx in the ProbeDealer package and can be customized by the users. The primary probe synthesis and amplification procedure with the template oligo library and the default primers will reverse complement the template oligo sequences to generate the primary probes^1,13^. If users require only the primary probe sequences and plan to order them directly, priming regions will not be added and oligo sequences (including the secondary regions) will be computationally reverse-complemented in the output files.

### Perform test runs with ProbeDealer

The following test runs in ProbeDealer was performed with a Dell Precision Tower 3630 workstation: five chromatin tracing test runs, which targeted: (1) 50 TADs on mouse chr19, (2) 19 consecutive 5-kb regions upstream of *Scd2* gene on mouse chr19, (3) 30 TADs along human chr20, (4) 34 TADs on human chr21, and (5) 40 TADs on human chrX; and one RNA MERFISH test run, which targeted 136 mouse transcripts. The workstation contained an Intel^®^ Core™ i7-8700 K CPU with 6 cores and 12 logical processors, and 32 GB RAM. One processor was used in all test runs.

For chromatin tracing test runs, the coordinates of targeted genomic region of mouse chr19 TADs and sequences upstream of *Scd2* gene locus were downloaded from Ref.^13^, and converted from mm9 to mm10 by the LiftOver tool in UCSC genome browser with default settings. Mouse mm10 genome assembly was used for both test runs. The genomic coordinates of human chr20, chr21 and chrX TADs were downloaded from Ref.^1^, and the central 100-kb region were used for probe design. For the three test runs targeting human genome, human hg18 genome assembly was used, instead of the default hg38 genome assembly. For all chromatin tracing test runs, we chose the chromatin tracing design scheme without additional features, and all probes were exported.

For MERFISH test runs, 137 mouse transcript IDs and RNA-seq FPKM values were acquired from Ref.^13^, and transcript IDs were converted from Gencode vM24 to Gencode vM25, with one failed transcript conversion. We chose the RNA MERFISH design scheme and exported all probes.

### Perform MERFISH test run with OligoArray 2.1

The 136 converted mouse transcripts were first divided into 1-kb fragments, and then supplied to OligoArray 2.1 with the following settings: probe length of 30 nucleotides, GC content between 30 and 90%, melting temperature above 66 °C, no 6 or more identical consecutive bases, no stable secondary structure above 76 °C, no cross-hybridization above 72 °C, and no overlaps between probes. OligoArray 2.1 was executed on Yale High Performance Computing Cluster with one node, one core and 32 GB RAM.

After probes were generated from OligoArray 2.1, probes were further BLASTed against mouse transcriptome Gencode vM25 with the same criteria as introduced in ProbeDealer MERFISH mode: probes targeting different transcript isoforms of the same gene were retained, while probes targeting multiple transcripts of different genes were rejected.

### Perform MERFISH test run with OligoMiner

The same 136 mouse transcripts were applied to OligoMiner blockParse.py with the following settings: probe length of 30 nucleotides, GC content between 30 and 90%, melting temperature above 66 °C, formamide concentration 0%, no 6 or more identical consecutive bases. The output probes were then supplied to structureCheck.py with the following settings: salt concentration 330 mM, hybridization temperature 37 °C. The probes that passed structureCheck were then BLASTed against transcriptome with the same criteria in MERFISH test run with OligoArray 2.1.

## Supplementary information

Supplementary Information 1.

Supplementary Information 2.

## Data Availability

All codes are available at https://campuspress.yale.edu/wanglab/ProbeDealer/.

## References

[1] Wang S (2016). Spatial organization of chromatin domains and compartments in single chromosomes. Science.

[2] Bintu, B. *et al.* Super-resolution chromatin tracing reveals domains and cooperative interactions in single cells. *Science* **362**, (2018). 10.1126/science.aau1783 PMC6535145 30361340

[3] Nir, G. *et al*. Walking along chromosomes with super-resolution imaging, contact maps, and integrative modeling. *PLoS Genetics* **14** (2018). 10.1371/journal.pgen.1007872 PMC6324821 30586358

[4] Mateo LJ (2019). Visualizing DNA folding and RNA in embryos at single-cell resolution. Nature.

[5] Cardozo Gizzi, A. M. *et al.* Microscopy-based chromosome conformation capture enables simultaneous visualization of genome organization and transcription in intact organisms. *Mol. Cell* **74**, 212–222.e5 (2019). 10.1016/j.molcel.2019.01.011 30795893

[6] Sawh, A. N. *et al.* Lamina-dependent stretching and unconventional chromosome compartments in early *C. elegans* embryos. *Mol. Cell* **78**, 96–111.e6 (2020). 10.1016/j.molcel.2020.02.006 PMC7263362 32105612

[7] Chen KH, Boettiger AN, Moffitt JR, Wang S, Zhuang X (2015). Spatially resolved, highly multiplexed RNA profiling in single cells. Science.

[8] Moffitt JR (2016). High-throughput single-cell gene-expression profiling with multiplexed error-robust fluorescence in situ hybridization. Proc. Natl. Acad. Sci. U. S. A..

[9] Moffitt, J. R. *et al.* Molecular, spatial, and functional single-cell profiling of the hypothalamic preoptic region. *Science* **362**, eaau5324 (2018). 10.1126/science.aau5324 PMC6482113 30385464

[10] Xia C, Fan J, Emanuel G, Hao J, Zhuang X (2019). Spatial transcriptome profiling by MERFISH reveals subcellular RNA compartmentalization and cell cycle-dependent gene expression. Proc. Natl. Acad. Sci. U. S. A..

[11] Lubeck E, Coskun AF, Zhiyentayev T, Ahmad M, Cai L (2014). Single-cell in situ RNA profiling by sequential hybridization. Nat. Methods.

[12] Eng CHL (2019). Transcriptome-scale super-resolved imaging in tissues by RNA seqFISH+. Nature.

[13] Liu M (2020). Multiplexed imaging of nucleome architectures in single cells of mammalian tissue. Nat. Commun..

[14] Beliveau BJ (2012). Versatile design and synthesis platform for visualizing genomes with Oligopaint FISH probes. Proc. Natl. Acad. Sci. U. S. A..

[15] Rouillard, J. M., Zuker, M. & Gulari, E. OligoArray 2.0: Design of oligonucleotide probes for DNA microarrays using a thermodynamic approach. *Nucleic Acids Res.* **31**, 3057–3062 (2003). 10.1093/nar/gkg426 PMC162330 12799432

[16] Camacho C (2009). BLAST+: architecture and applications. BMC Bioinformatics.

[17] Beliveau BJ (2018). OligoMiner provides a rapid, flexible environment for the design of genome-scale oligonucleotide in situ hybridization probes. Proc. Natl. Acad. Sci. U. S. A..

[18] Quinlan AR, Hall IM (2010). BEDTools: a flexible suite of utilities for comparing genomic features. Bioinformatics.

[19] SantaLucia J (1998). A unified view of polymer, dumbbell, and oligonucleotide DNA nearest-neighbor thermodynamics. Proc. Natl. Acad. Sci. U. S. A..

