# Supplementary material for: ProbeDealer is a convenient tool for designing probes for highly multiplexed fluorescence in situ hybridization
Source: Sci Rep. 2020 Dec 16;10:22031. doi: 10.1038/s41598-020-76439-x (PMC7745008; doi:10.1038/s41598-020-76439-x)
Supplement: Supplementary file 1 — Supplementary Information 1. [file 41598_2020_76439_MOESM1_ESM.pdf]

Supplementary Information for:

ProbeDealer is a convenient tool for designing probes for highly multiplexed fluorescence *in situ* hybridization

Authors: Mengwei Hu[1#], Bing Yang[1#], Yubao Cheng[1], Jonathan S. D. Radda[1], Yanbo Chen[1], Miao Liu[1] and Siyuan Wang[1,2,3,4,5,6,7,8*]

Affiliations: [1]Department of Genetics, [2]Department of Cell Biology, [3]Yale Combined Program in the Biological and Biomedical Sciences, [4]Molecular Cell Biology, Genetics and Development Program, [5]Biochemistry, Quantitative Biology, Biophysics and Structural Biology Program, [6]M.D.-Ph.D. Program, [7]Yale Center for RNA Science and Medicine, [8]Yale Liver Center, Yale University School of Medicine, New Haven, CT 06510, USA

*Correspondence to: siyuan.wang@yale.edu

#These authors contributed equally to this work.

Supplementary Note 1: ProbeDealer user manual

1. Install BLAST

Windows users should download and install ncbi-blast-2.10.1+-win64.exe from https://ftp.ncbi.nlm.nih.gov/blast/executables/blast+/LATEST/. Mac users should download and install ncbi-blast-2.10.1+.dmg from https://ftp.ncbi.nlm.nih.gov/blast/executables/blast+/LATEST/. Mac users then need to add the BLAST executable bin file to the PATH by following these instructions:

In Terminal, type: sudo nano /etc/paths
enter password
scroll down to the bottom of the list
add the path: /usr/local/ncbi/blast/bin
exit and save the list
restart the terminal

Mac users may use the following command in Terminal to verify BLAST installation:

type: echo $PATH
expected outcome:
/usr/local/bin:/usr/bin:/bin:/usr/sbin:/sbin:/usr/local/ncbi/blast/bin
type: which blastn
expected outcome: /usr/local/ncbi/blast/bin/blastn

2. Install ProbeDealer

ProbeDealer provides MATLAB add-on applications for Windows and Mac users and a standalone application for Windows users, available for download from https://campuspress.yale.edu/wanglab/probedealer.

To install the ProbeDealer MATLAB application, open MATLAB, and click "Install App" in APP tab. Select a .mlappinstall file according to your operating system. To use the ProbeDealer standalone application on Windows, install MATLAB Runtime version 9.8 (R2020a), and double click ProbeDealer_Win.exe to execute the standalone application.

Users of ProbeDealer MATLAB application need to install the MATLAB Bioinformatic Toolbox in order to use ProbeDealer.

3. ProbeDealer usage

3.1 Provide BLAST genome database

Input path to genome file folder containing necessary BLAST database subfolders. This path should not contain spaces.

For chromatin tracing probes, provide the genome file folder that contains the following sub-folder: "genome", for the genome BLAST database. To enable the "only target antisense of genes" feature, also include a sub-folder "UnsplicedTx" as the unspliced

transcriptome database. To enable the "avoid exon regions" feature, include another sub-folder "TxShortHeader" as the transcriptome database.

For sequential single-molecule RNA FISH and MERFISH probes, provide the genome file folder path that contains the following sub-folders: "TxShortHeader", as the transcriptome database, and "Tx", which contains the Gencode transcriptome fasta file with full headers to match transcripts with their genes. Note that for all probe types, the sub-folders should be named as indicated in this manual.

We provide example "genome", "UnsplicedTx", "TxShortHeader", and "Tx" subfolders that contain human and mouse databases at https://campuspress.yale.edu/wanglab/probedealer. Users may download these two folders, and use the folder paths as inputs for ProbeDealer to design human and mouse probes.

For additional user-defined databases, we recommend using UCSC genome fasta files to generate BLAST databases and UCSC GTF files to generate unspliced transcriptomes. ProbeDealer only accepts Gencode transcriptome files.

3.2 Choose probe type

Select a probe type in the application panel. Chromatin tracing users may choose additional features according to their needs. Users may also specify their preferred oligo parameters by editing oligoparameters.xlsx provided in the ProbeDealer package. For

example, to allow designed probes to overlap each other by up to 20 nucleotides, as previously demonstrated in chromatin tracing to trace a gene *cis*-regulatory region at 5-kb genomic resolution[3], users may change ProbeGap in oligoparameters.xlsx from 31 to 11. This overlapping design allows more probes to be designed for short genomic regions or RNA species.

oligoparameter.xlsx includes the following parameters that can be customized:

ProbeLength: desired length of oligos. Default is 30 nt.

MinTm: minimum Tm of oligos. Default is 66 °C.

MaxTm: maximum Tm of oligos. Default is 100 °C.

SecondaryStructureTm: maximum Tm of concatenated stems in the oligo. Default is 76 °C.

CrossHybTm: maximum Tm of concatenated cross-hybridization regions in the oligo. Default is 72 °C.

MinGC: minimum percentage of GC of oligos. Default is 30%.

MaxGC: maximum percentage of GC of oligos. Default is 90%.

ExcludeSeq: sequences that should be avoided in oligos; excluded sequences are separated by "|". Default is GGGGGG|CCCCCC|TTTTTT|AAAAAA.

ProbeGap: minimum distance between the 5' end of two adjacent oligos. Default is 31, for generating oligos of 30-nt with no overlap.

3.3 Provide target sequences

ProbeDealer accepts two target input file formats: fasta file (.fasta, .fa, .fas) and spreadsheet file (*.xls, *.xlsx, *.csv). Chromatin tracing and sequential single-molecule RNA FISH accept both types of input, while MERFISH only accepts spreadsheet files. Example target input files are available for download from https://campuspress.yale.edu/wanglab/probedealer.

The spreadsheet file for chromatin tracing should contain three columns. The first column indicates the chromosome of current target sequence, e.g. chr1 (note that "chr" should be present to be consistent with the format of the UCSC genome fasta file). The second and third columns indicate the start and end points of the target sequence on the indicated chromosome, and coordinates should start from 1, inclusive (i.e. same as UCSC GTF coordinates, not UCSC BED coordinates). As a simplified example, for a sequence on a chromosome:

>chr1
ATCTATTTGGGCG

To design chromatin tracing probe for TATTT, the three columns should be:

chr1   4   8

To use our default human and mouse databases, the genome coordinates need to be from hg38 and mm10, respectively.

For RNA FISH, ProbeDealer will draw target information from the first column of the input spreadsheet. The first column should contain the Ensembl transcript IDs (without version) of target sequences (e.g. ENST00000544455, ENSMUST00000106216).

The spreadsheet file for RNA MERFISH should contain at least two columns. The first column should contain the Ensembl transcript IDs (without version) of target sequences, and the second column should contain the corresponding gene FPKM values or other measures of relative transcript expression levels.

Users should ensure the number of input sequences does not exceed the number of available secondary sequences (or secondary sequence combinations in MERFISH) in the provided spreadsheets, DNA secondaries.xlsx (for chromatin tracing probes) and RNA secondaries.xlsx (for sequential single-molecule RNA FISH and MERFISH). By default, we provide 50 secondary sequences for chromatin tracing and 16 secondary sequences for sequential single-molecule RNA FISH. Thus, the chromatin tracing target number should be $\leq$ 50, and the sequential single-molecule RNA FISH target number should be $\leq$ 16. MERFISH accepts up to 140 target sequences.

Users may also specify how many probes they want for each input sequence, or choose to retain all probes. If an input sequence does not have as many probes as specified by the user, some of its probes will be duplicated to meet the requirement. We recommend at least 48 probes for each RNA target in MERFISH, at least 36 probes for each RNA target

in sequential single-molecule RNA FISH, and at least 150 probes for each genomic target in chromatin tracing[3].

3.4 Output

Choose output type and provide a path for output files. Output files include FinalOligos.fasta and FinalOligos.xlsx. If some sequences do not have as many probes as specified by the user, a log file will be generated to record those input sequences. A codebook will be generated as an Excel spreadsheet for MERFISH users to match each transcript with its MHD4 code.

Supplementary Notes 2: Ordering dye-labeled secondary probes and primers

To visualize genomic loci or RNA foci in experiment, users should purchase dye-labeled secondary probes with fluorescent dyes appended to the 5’ end of the sequences included in the DNA secondaries.xlsx and RNA secondaries.xlsx files provided with the ProbeDealer package, respectively. Examples of the 50 secondary probes for chromatin tracing were reported in Liu *et al* Supplementary Data File 6 in the “TAD tracing” tab[1]. Examples of the 16 secondary probes for RNA MERFISH were reported in Liu *et al* Supplementary Data File 6 in the “RNA MERFISH” tab[1].

To synthesize and amplify primary probes from a template oligo library, users should order primers according to the sequences listed in Primers.xlsx. When ordering forward primers, users should append the T7 promoter sequence (GCCGTACGGATAATACGACTCACTATAGGG) at the 5’ end of forward primers. When ordering the reverse primers, users should reverse-complement the reverse priming sequences in Primers.xlsx. The primers to order for the three default pairs of priming sequences are: For chromatin tracing, forward primer: GCCGTACGGATAATACGACTCACTATAGGG GTGGTAAAGCTCCGCGGCTT; reverse primer: TCGTTCCGCATTGACCAATC. For RNA MERFISH, forward primer: GCCGTACGGATAATACGACTCACTATAGGG CCCGCGTTAACCATACACCG; reverse primer: CATCGAAGCGTGTGGCTACC. For sequential single-molecule RNA FISH, forward primer: GCCGTACGGATAATACGACTCACTATAGGG GCGTCGTTATGGTGCAACGT; reverse primer: TTGTCGCACGTTCGGTGTCG.

Supplementary Note 3: Additional comparison between ProbeDealer and other probe design packages

We compared ProbeDealer with other available probe design tools, including OligoArray2.1[2], a MATLAB package for RNA MERFISH design[3] and OligoMiner[4]. We compare these tools on the following aspects:

1) Purpose of the software. Unlike the other tools, ProbeDealer is intended for designing probes for a variety of multiplexed FISH implementations, including Multiplexed Imaging of Nucleome Architectures (MINA), multiscale chromatin tracing, RNA MERFISH and sequential single-molecule RNA FISH, which involve multiple unique probe design schemes and considerations. In particular, the primary probe sequences need to be specific in targeting genomic loci or transcripts of interest in chromatin tracing and RNA MERFISH implementations, respectively; and when RNA MERFISH and chromatin tracing are combined in the MINA implementation, the primary probes targeting genomic loci and RNA species must not interfere with each other (competing for the same target or cross-hybridize with each other). To meet these requirements, we implemented BLAST algorithms that are most suitable for each different probe design scenario. In addition, for the convenience of users, ProbeDealer provides a set of tested secondary probe sequences that are orthogonal to the human and mouse genomes[1,3] to be automatically used in primary probe designs. For RNA MERFISH, ProbeDealer also automatically includes the repeatedly tested MERFISH codebook with a Modified Hamming Distance 4 (MHD4) coding scheme [1,3,5–9]. OligoArray 2.1 and OligoMiner generate probes for primary FISH and can be used for both DNA and RNA FISH,

however, they do not include the steps of adding secondary and priming sequences for the multiplexed FISH implementations. The RNA MERFISH MATLAB package is specialized in designing RNA MERFISH probes, but not chromatin tracing probes. Therefore, ProbeDealer is the most convenient and versatile tool in designing probes for a variety of multiplexed DNA and RNA FISH implementations.

2) Computational resource requirements and time cost. Based on our tests, ProbeDealer can be executed on both a workstation with 32 GB RAM and a laptop with 16 GB RAM, although the latter takes slightly longer time to finish the design especially when designing probes for tens of long (100 kb) target sequences. ProbeDealer can typically finish designing probes for fine-scale chromatin tracing (*e.g.* targeting tens of 5-kb genomic regions) and MERFISH of 140 transcripts within half an hour; when designing probes for long sequences, including whole chromosome tracing probes which typically target tens of 100-kb regions, ProbeDealer can finish the design within one day on a workstation. OligoArray 2.1 is no longer available for download, which prevents us from testing it on our workstation. The existing copy of OligoArray 2.1 available to us is installed on the Yale High Performance Computing Cluster. By requesting 32 GB of RAM and one core from the cluster, we tested the time it takes to run the same MERFISH probe design targeting 136 mouse transcripts. OligoArray 2.1 finished the probe generating step in 1.9 hours, which did not include other crucial steps, including MHD4 codebook design and probe specificity test. ProbeDealer finished the same probe design procedure in 18.5 minutes. Thus, we conclude that ProbeDealer is significantly faster than OligoArray 2.1. The RNA MERFISH MATLAB package is recommended to

run with 32 GB to 64 GB RAM (github.com/ZhuangLab/MERFISH_analysis). The time cost of the package can reach several hours on a workstation with parallel computing to systematically profile the transcriptome and generate oligos that may be used as part of the primary probes. However, once this step is finished, such profile can be repeatedly used in different probe design tasks, and the package can generate probes with 5-10 minutes[3]. OligoMiner is capable of designing probes for the whole human genome within hours[4], which can be executed on a personal laptop with 16 GB RAM.

3) Probe design criteria. All the tools allow customizable probe length, GC content and melting temperature. ProbeDealer, OligoArray 2.1 and OligoMiner consider the secondary structure and cross-hybridization effects and reject the probes that do not pass the secondary structure or cross-hybridization tests. All the tools evaluate probe specificity to eliminate off-target effect. ProbeDealer, OligoArray 2.1 and OligoMiner adopt alignment tools to identify off-target binding. ProbeDealer and OligoArray 2.1 uses BLAST, and OligoMiner uses Bowtie2 for faster alignment. The RNA MERFISH MATLAB package constructs isoform penalty tables and transcriptome penalty table by analyzing 17-nucleotide-long sequences for potential off-target events.

4) User-friendliness. ProbeDealer is the only one among the four tools that offers a graphical-user interface on a local computer, and users can easily customize the probe design parameters by editing an Excel spreadsheet that stores those parameters. OligoArray 2.1 and OligoMiner are both executed via command line operations. The RNA MERFISH MATLAB package consists of MATLAB scripts and functions, which

may be challenging for user without coding experience to customize the probe design process to best suit their research needs. Therefore, OligoArray 2.1, OligoMiner and the RNA MERFISH MATLAB package may be less preferred by users with limited coding expertise.

References:

1. Liu, M. *et al.* Multiplexed imaging of nucleome architectures in single cells of mammalian tissue. *Nat. Commun.* **11**, 1–14 (2020).
2. Rouillard, J. M., Zuker, M. & Gulari, E. OligoArray 2.0: Design of oligonucleotide probes for DNA microarrays using a thermodynamic approach. *Nucleic Acids Res.* **31**, 3057–3062 (2003).
3. Moffitt, J. R. *et al.* High-throughput single-cell gene-expression profiling with multiplexed error-robust fluorescence in situ hybridization. *Proc. Natl. Acad. Sci. U. S. A.* **113**, 11046–11051 (2016).
4. Beliveau, B. J. *et al.* OligoMiner provides a rapid, flexible environment for the design of genome-scale oligonucleotide in situ hybridization probes. *Proc. Natl. Acad. Sci. U. S. A.* **115**, E2183–E2192 (2018).
5. Chen, K. H., Boettiger, A. N., Moffitt, J. R., Wang, S. & Zhuang, X. Spatially resolved, highly multiplexed RNA profiling in single cells. *Science* **348**, 1360–1363 (2015).

6. Moffitt, J. R. *et al.* High-performance multiplexed fluorescence in situ hybridization in culture and tissue with matrix imprinting and clearing. *Proc. Natl. Acad. Sci. U. S. A.* **113**, 14456–14461 (2016).

7. Moffitt, J. R. *et al.* Molecular, spatial, and functional single-cell profiling of the hypothalamic preoptic region. *Science* **362**, eaau5324 (2018).

8. Wang, G., Moffitt, J. R. & Zhuang, X. Multiplexed imaging of high-density libraries of RNAs with MERFISH and expansion microscopy. *Sci. Rep.* **8**, 1–13 (2018).

9. Xia, C., Babcock, H. P., Moffitt, J. R. & Zhuang, X. Multiplexed detection of RNA using MERFISH and branched DNA amplification. *Sci. Rep.* **9**, 1–13 (2019).
